# Baseline Characteristics Associated With Sodium-Glucose Cotransporter Inhibitor Prescriptions in Type 2 Diabetic Patients in Jazan, Saudi Arabia

**DOI:** 10.7759/cureus.24284

**Published:** 2022-04-19

**Authors:** Mohammed Somaili, Omar Oraibi, Mostafa Mohrag, Abdelrahman Hommadi, Esam Moafa, Abdulrahman Kulaybi, Sahar Shobayli, Razan Moafa, Ghadah Mhgfory, Afaf Jaafari, Ayman Shami, Khalid Majrashi

**Affiliations:** 1 Internal Medicine, Jazan University, Jazan, SAU; 2 Endocrinology, Jazan University, Jazan, SAU; 3 Endocrinology, Ministry of Health Holdings, Jazan, SAU; 4 Medicine and Surgery, Jazan University, Jazan, SAU

**Keywords:** saudi arabia, jazan, type 2 diabetes mellitus, sodium glucose co-transporter inhibitors, baseline characteristics

## Abstract

Background: Sodium-glucose cotransporter inhibitors are promising medications for improving cardiovascular outcomes in diabetic and non-diabetic patients. The baseline characteristics associated with its prescription in Jazan, Saudi Arabia, are still lacking.

Objectives: This study aims to determine the baseline characteristics associated with its prescription among type 2 diabetic patients in the Jazan region.

Methods: A retrospective cohort study of adult patients with type 2 diabetes mellitus (T2DM) in Jazan region, Saudi Arabia, who received a prescription of sodium-glucose cotransporter 2 inhibitors (SGLT2i) from June 2016 to December 2021 was conducted. Categorical baseline characteristics are reported as frequencies, and proportion and continuous variables are reported as means (SD). The crude odds and adjusted ratio (OR) (95% CI) for SGLT2i prescription were then calculated using univariate and multivariate logistic regression analysis.

Results: A total of 370 patients who satisfied the inclusion criteria were included in this study. There were 133 patients (36%) who had been prescribed SGLT2i over a median follow-up of five years. Characteristics associated with the prescription were female gender (adjusted odds ratio [aOR]: 2.2, 95% Cl: 1.3-3.9), endocrinologist doctors (aOR: 2.4, 95% Cl: 1.3-4.5), patients who had visited the center four times or more (aOR: 10.9, 95% Cl: 4.6-25.9), patients who have evidence of DM retinopathy (aOR: 9.7, 95% Cl: 2.9-31.7), or patients who are morbidly obese (aOR: 4.1, 95% Cl: 1.1-14.8).

Conclusion: The sodium-glucose cotransporter inhibitors are under-prescribed among type 2 diabetic patients in the Jazan region despite their availability. Further studies are warranted to address the potential barriers to prescriptions among different medical specialties.

## Introduction

Type 2 diabetes mellitus (T2DM) is a major health problem worldwide and is associated with increased morbidity and mortality [1,2]. The prevalence of T2DM is increasing, with a global prevalence of 426 million individuals being affected by this disease, and attributed to approximately one million deaths per year [3,4].

Fortunately, several oral and injectable antidiabetic medications with different mechanisms of action have been developed to control hyperglycemia and mitigate the associated risks [5-8]. One of these classes is the sodium-glucose cotransporter 2 inhibitors (SGLT2i). The current available SGLT2i are canagliflozin, empagliflozin, dapagliflozin, and ertugliflozin. These medications have recently been introduced into clinical practice to help attain optimal glycemic control. They work by inhibiting the reabsorption of filtered glucose through sodium-glucose transporters located in the proximal convoluted tubules of kidney nephrons [9]. Their role in patients with type 2 diabetes extends beyond just being an antidiabetic medication.

Recent data have shown that these agents have a renoprotective effect and better cardiovascular and mortality outcomes [10-12]. The Canagliflozin and Renal Events in Diabetes with Established Nephropathy Clinical Evaluation (CREDENCE) trial, for instance, showed favorable outcomes of canagliflozin in slowing the progression of diabetic kidney disease (DKD) among T2DM as well as major cardiovascular events. These favorable outcomes have also been reported with empagliflozin and dapagliflozin in the Empagliflozin Cardiovascular Outcome Event Trial in Type 2 Diabetes Mellitus Patients: Removing Excess Glucose (EMPA-REG) and Dapagliflozin and Prevention of Adverse Outcomes in Chronic Kidney Disease (DAPA-CKD), respectively [10,11,13-15].

Despite these positive outcomes and strong guideline recommendations [16-18], physicians prescribing these medications remains low [19]. For example, a recent study revealed that the prescription rate is as low as 3%, particularly among cardiologists, even after the US Food and Drug Administration expanded the labeling of these drugs [19,20].

In Jazan, a small region located in the southern part of Saudi Arabia, health services are growing and escalating in harmonized patterns to catch up with other regions in the kingdom, aiming to provide the best available health services [21]. An observational study from the Jazan Diabetic Center recently showed that more than 85% of patients managed at this center had suboptimal diabetic control with HbA1c > 7%. However, this study did not highlight or address the oral hypoglycemic classes used to achieve glycemic control in the study cohorts [22]. In our study, we aim to assess the baseline characteristics of SGLT2i prescription among patients with type 2 diabetes in Jazan Diabetic Center, Saudi Arabia.

## Materials and methods

Study objective

This study aims to determine the baseline characteristics associated with sodium-glucose cotransporter-2 inhibitor (SGLT2i) prescription among T2DM patients in the Jazan region of Saudi Arabia.

Study design

This was a retrospective cohort study of adult patients with T2DM in the Jazan region of Saudi Arabia who received an SGLT2i prescription from June 2016 to December 2021.

Study setting

The study was conducted at the Jazan Diabetic Center. The center was established in 2011 as an outpatient-based facility that serves approximately 1,000 and 9,400 patients with type 1 and 2 diabetes, respectively. The center is equipped with all laboratory and radiological investigations to serve patients with diabetes or its complications. In addition, all oral and injectable diabetes medications, including SGLTi, with some variability in the availability of individual types, are available at this center.

Study cohorts

All adult patients with T2DM who were followed up in this center were included in this study. Patients younger than 14 years of age or those who died on or before the index date were excluded.

Data sources

Data were obtained from physical charts. These data include patient characteristics, duration of diabetes, comorbidities, year of prescription, name of SGLT2i prescribed, sole or add-on SGLT2i prescription, and prescribing physician specialty. Laboratory data included glycosylated hemoglobin (HbA1c) and estimated glomerular filtration rate (eGFR) at baseline and different follow-up intervals.

Patients’ comorbidities and healthcare utilization were assessed five and two years before the index date (the date of SGLT2i introduction into Saudi Arabia, June 2016). Laboratory data were assessed at baseline and three-, six-, and nine-month intervals after prescription. Medications were assessed three months before the index date.

Sample size estimates and sampling technique

Based on the total number of patients with type 2 diabetes visiting the center (n = 9,400), the estimated sample size was 370 with a 95% confidence interval and a marginal error of 0.05. A simple random sampling technique was used, in which 370 patients with type 2 diabetes were screened and then followed up until they received the outcome of interest (SGLT2i prescription).

Data extraction technique

After obtaining approval from the Jazan Health Ethics Committee, the researchers visited the center and collected data in an Excel spreadsheet according to the prespecified variables.

Statistical analysis

Categorical baseline characteristics are reported as frequencies, proportions, and continuous variables are reported as means (SD). Standardized differences were used to compare baseline characteristics between SGLT2i users and non-users with a standardized difference of >0.1, which is considered a significant difference between the two groups. The patients were followed up until the first SGLT2i prescription (outcome), censoring event (death or loss of follow-up), or end of follow-up (December 2021). The prescription will be examined at baseline (2016) and then in the following five years (2017-2021). Crude odds and adjusted ratio (OR) (95% CI) for SGLT2i prescription were calculated using univariate and multivariate logistic regression analyses. A two-sided p-value of <0.05 is considered an indicator of statistical significance. All analyses were conducted using Statistical Package for the Social Sciences (SPSS), version 26 (IBM Corp., Armonk, NY).

## Results

Baseline characteristics

A total of 370 patients who satisfied the inclusion criteria were included in this study. Most of the patients with diabetes were middle-aged (40-60 years), and most were women (53%), most of whom had DM for five to 10 years (53%), and the majority of them had no DKD (93%). Hypertension was the most common comorbidity (52%). Fifty-one percent had three to four visits to the DM center in the last two years, while 30% had more than four visits. SGLT2i prescription was started as soon as 2018 in this center with empagliflozin being the most prescribed (Table 1).

**Table 1:** Basic characteristics of patients with type 2 diabetes according to SGLT2i prescription ^#^p-value is based on Pearson's chi-squared test. *p-value is based on Fisher's exact test. Data are presented as the number (percentage) of individuals. T2DM = Type two diabetes mellitus; eGFR = Estimated glomerular filtration rate; DKD = Diabetic kidney disease; DLP = Dyslipidemia; HTN = Hypertension; SGLT2 = sodium-glucose cotransporter 2; CI = Confidence interval.

Characteristics		Total	Prescription of SGLT2i		p-value^#^
			No	Yes	
		N%	N%	N%	
Gender	Male	175 (47.3)	127 (72.6)	48 (27.4)	0.001
	Female	195 (52.7)	110 (56.4)	85 (43.6)	
Age groups	18-40 years	40 (10.8)	28 (70.0)	12 (30.0)	0.002
	41-60 years	175 (47.3)	96 (54.9)	79 (45.1)	
	>60 years	155 (41.9)	113 (72.9)	42 (27.1)	
Nationality	Saudi	346 (93.5)	220 (63.6)	126 (36.4)	0.474
	Non-Saudi	24 (6.5)	17 (70.8)	7 (29.2)	
Duration of T2DM	<5 years	16 (4.3)	12 (75.0)	4 (25.0)	0.165
	5-10 years	196 (53.0)	132 (67.3)	64 (32.7)	
	>10 years	158 (42.7)	93 (58.9)	65 (41.1)	
Presence of DKD	No	345 (93.2)	216 (62.6)	129 (37.4)	0.031
	Yes	25 (6.8)	21 (84.0)	4 (16.0)	
Physician specialty	Internal medicine	25 (6.8)	8 (32.0)	17 (68.0)	<0.001
	Endocrinologist	89 (24.1)	45 (50.6)	44 (49.4)	
	Family physician	230 (62.2)	159 (69.1)	71 (30.9)	
	GP	26 (7.0)	25 (96.2)	1 (3.8)	
Visits to diabetic center	1-2	61 (16.5%)	48 (78.7)	13 (21.3)	<0.001
	3-4	189 (51.2)	148 (78.3)	41 (21.7)	
	4 and more	119 (32.2)	40 (33.6)	79 (66.4)	
eGFR	eGFR > 60	345 (93.2)	216 (62.6)	129 (37.4)	0.076*
	eGFR = 30-60	21 (5.7)	17 (81.0)	4 (19.0)	
	eGFR < 30	4 (1.1)	4 (100.0)	0 (.0)	
Comorbidities	DLP	6 (1.6)	3 (50.0)	3 (50.0)	0.152*
	DM neuropathy	20 (5.4)	11 (55.0)	9 (45.0)	
	DM retinopathy	35 (9.5)	17 (48.6)	18 (51.4)	
	HTN	192 (51.9)	123 (64.1)	69 (35.9)	
	IHD	15 (4.1)	11 (73.3)	4 (26.7)	
	Morbid obesity	25 (6.8)	14 (56.0)	11 (44.0)	
	Other	9 (2.4)	6 (66.7)	3 (33.3)	
	None	68 (18.4)	52 (76.5)	16 (23.5)	
Overall incidence of use		370 (100)	237 (64.1)	133 (35.9), 95% CI: 31.2-41.0	

Use of SGLT2i

**Figure 1:**
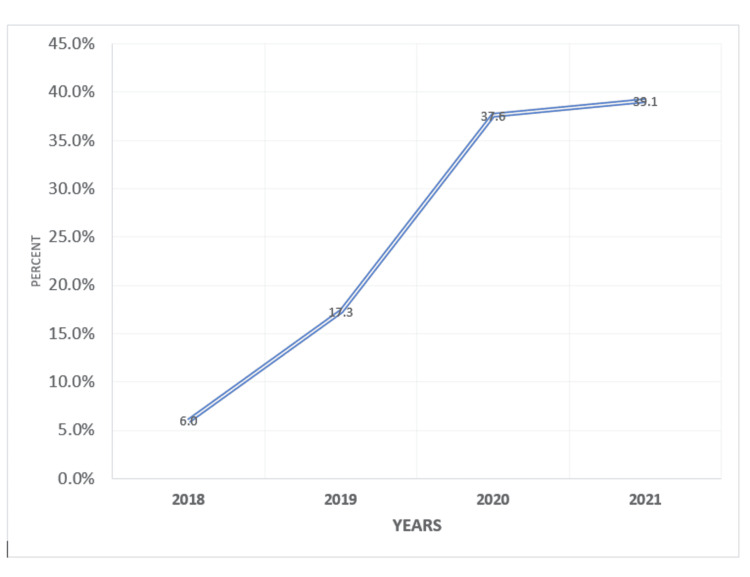
Sodium-glucose cotransporter 2 inhibitor use among study participants during the period 2018-2021

A total of 133 patients (36%) were prescribed SGLT2i over a median follow-up period of five years. When examined by era, the incidence rate of SGLT2i prescriptions was 0% at baseline and 0%, 6%, 17%, 37%, and 39% in 2017, 2018, 2019, 2020, and 2021, respectively (Figure 1).

Patients’ characteristics associated with SGLT2i prescription

The patient characteristics of SGLT2i vs. non-SGLT2i are outlined in Table 1. SGLT2i users were mainly females (44%), more likely aged 41-60 years, of Saudi nationality, whose DM duration was > five years (74%), and had no evidence of DKD. In addition, patients with four visits or more (79%), with a history of hypertension, morbid obesity, or evidence of DM retinopathy, were more likely to receive the prescription. The most prescribers of SGLT2i were the endocrinologists (49%) followed by family physicians (31%).

When compared using multivariate logistic regression analysis, female sex (adjusted odds ratio [aOR]: 2.2, 95% Cl: 1.3-3.9), endocrinologist prescribers (aOR: 2.4, 95% Cl: 1.3-4.5), patients who had visited the center four times or more (aOR: 10.9, 95% Cl: 4.6-25.9), evidence of DM retinopathy (aOR: 9.7, 95% Cl: 2.9-31.7), or morbid obesity (aOR: 4.1, 95% Cl: 1.1-14.8) were associated with higher SGLT2i prescription. Patients older than 60 years (aOR: 0.17, 95% Cl: 0.48-1.34), patients with evidence of DKD (aOR: 0.37, 95% Cl: 0.11-1.26), and general practitioner prescribers (aOR: 0.05, 95% Cl: 0.01-0.46) were associated with lower SGLT2i prescription (Table 2).

**Table 2:** Univariate and multivariate logistic regression analyses of factors associated with SGLT2 inhibitor use among all patients in Jazan *Reference category ^#^Adjusted for other variables in the table T2DM = Type two diabetes mellitus; DKD = Diabetic kidney disease; DLP = Dyslipidemia; HTN = Hypertension; SGLT2 = Sodium-glucose cotransporter 2; CI = Confidence interval; cOR = Crude odds ratio; aOR = Adjusted odds ratio.

Category	Univariate				Multivariate^#^			
	p-value	cOR	95% CI		p-value	aOR	95% CI p-value	
			Lower	Upper			Lower	Upper
Gender								
Male*		1						
Female	0.001	2.04	1.32	3.16	0.005	2.20	1.26	3.83
Age groups								
18-40 years*		1						
41-60 years	0.084	1.92	0.92	4.02	1.38	0.53	3.60	1.38
>60 years	0.715	0.87	0.40	1.86	0.48	0.17	1.34	0.48
Duration of diabetes								
<5 years*								
5-10 years	0.530	1.46	0.45	4.69	2.28	0.51	10.25	2.28
>10 years	0.217	2.10	0.65	6.79	3.76	0.77	18.35	3.76
Presence of DKD								
No*		1						
Yes	0.040	0.32	0.11	0.95	0.111	0.37	0.11	1.26
Physician specialty								
Family physician*		1						
Endocrinologist	0.002	2.19	1.33	3.61	0.005	2.42	1.31	4.48
Internal medicine	0.001	4.76	1.96	11.54	0.010	3.79	1.38	10.43
GP	0.019	0.09	0.01	0.67	0.007	0.05	0.01	0.46
Visits to diabetic center								
1-2*		1						
3-4	0.950	1.02	0.51	2.07	0.286	1.56	0.69	3.53
4 and more	0.000	7.29	3.55	15.00	0.000	10.88	4.60	25.75
Comorbidities								
None*		1						
DLP	0.166	3.31	0.61	18.04	0.278	3.10	0.40	23.88
DM neuropathy	0.061	2.71	0.95	7.69	0.074	3.49	0.89	13.70
DM retinopathy	0.005	3.51	1.47	8.35	0.000	9.67	2.95	31.72
HTN	0.052	1.87	1.00	3.53	0.160	1.87	0.78	4.50
IHD	0.775	1.20	0.34	4.30	0.242	2.51	0.54	11.78
Morbid obesity	0.053	2.60	0.99	6.85	0.026	4.19	1.19	14.80
Other	0.508	1.66	0.37	7.38	0.435	2.08	0.33	13.02

Glycemic control associated with the use of SGLT2i

**Figure 2:**
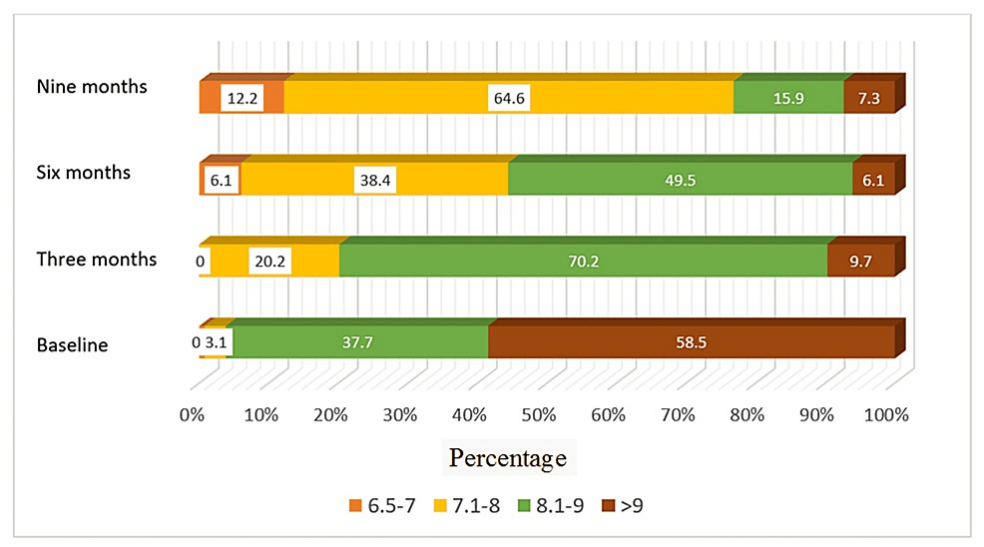
Distribution of HbA1C for patients with sodium-glucose cotransporter 2 inhibitors during four time periods after implementation

**Figure 3:**
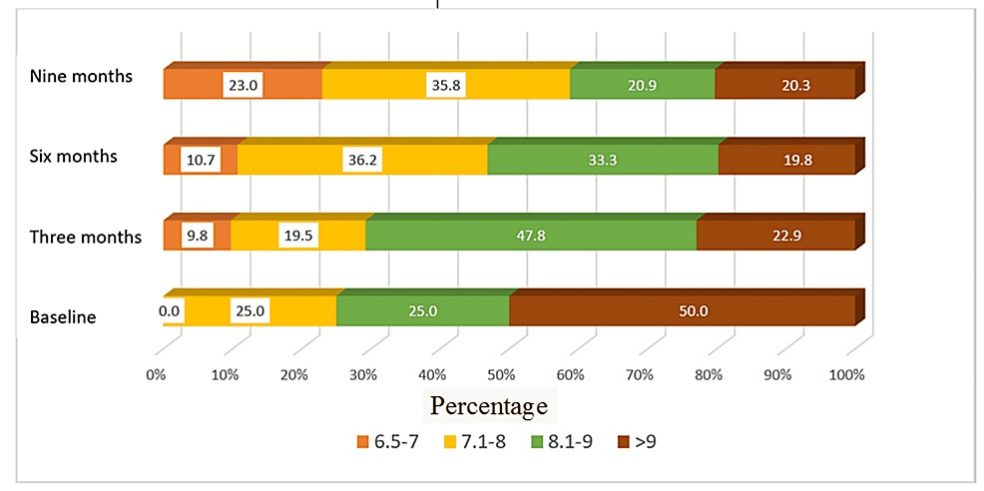
Distribution of HbA1c for patients without sodium-glucose cotransporter 2 inhibitors during four time periods

In terms of glycemic control, HbA1c at baseline was 6.5-7, 7.1-8, 8.1-9, and more than 9 in 0%, 3%, 38%, and 59%, respectively, among SGLT2i users and 0%, 25%, 25%, and 50%, respectively for non-SGLT2i users. After nine months of follow-up, the HbA1c readings were 6.5-7, 7.1-8, 8.1-9, and more than 9 in 12%, 65%, 16%, and 7%, respectively, in SGLT2i users and 23%, 36%, 21%, and 20% of non-SGLT2i users, respectively (Figures 2, 3).

## Discussion

In this retrospective cohort study of 370 patients with diabetes, we found that 36% of patients had SGLT2i prescription, while 64% did not, indicating that SGLT2i are still under-prescribed despite the favorable morbidities and mortality profile. We also found that the rate of SGLT2i prescription increased over time; it increased from 0% to 39% over a five-year period, which may increase awareness among physicians over time due to expanding evidence in this area. The most common factors associated with SGLT2i were female sex, middle age, longer duration of DM, absence of evidence of DKD, higher comorbidity index, four or more visits to the diabetic center, and an endocrinologist being the prescriber.

Glycemic control was very poor (>9%) at baseline in SGLT2i and non-SGLT2i users. It improved significantly after nine months of SGLT2i prescription, as added to the usual DM medications (50% and 56% vs. 7% and 20%, respectively). Despite the introduction of SGLTi into Saudi Arabia and approval by the Saudi Food and Drug Administration in 2016 [23], the physicians in this center, including endocrinologists, have just started the prescription in 2018. The low rate of SGLT2i prescription was in concordance with other studies that also showed infrequent prescriptions (17%) of this class of medication in clinical practice [24]. The potential barriers underlying this low rate include lack of physician familiarity with this class of diabetic medications, their cost compared with other antidiabetics, a transient decrease in renal function, and clinical inertia [25,26].

Clinical inertia usually leads to a delay in adopting a new class of medications to manage chronic diseases. Therefore, it could represent the strongest factor for the low prescription rate of SGLT2i among patients with type 2 diabetes [25]. Fortunately, the prescription rate has increased over time in this cohort. This finding is similar to a study by Hofer et al. [27]. The increased rate of SGLT2i prescription in our cohort parallels the strong recommendations to use this class of medications in patients with diabetes with or without cardiovascular risk factors due to their favorable outcomes in risk reduction [20,24,28].

Our study also showed that endocrinologists were the most common prescribers among the four specialties available in this center (general practitioners, certified family physicians, internists, and endocrinologists). This can be clearly explained by their direct involvement in the management of patients with diabetes, and their broad knowledge of DM management guidelines and literature. However, despite being the primary specialty involved in cardiovascular disease management, cardiologists are among the lowest prescribers of SGLT2i. This finding was endorsed by Dava et al., who found that the prescription barriers were the belief of cardiologists that they should not prescribe it and their lack of knowledge of these medications [20,29].

Among those who received the prescription, the vast majority (93%) had no evidence of chronic kidney disease, and only 7% of patients had an eGFR of 30-60 ml/min/1.732. This finding is aligned with guideline recommendations that state that the cardiovascular and renal protective effects of SGLT2i remain until kidney function severely drops (eGFR < 30 ml/min/1.732) [30-32]. The possible reason for the low prescription rate among CKD patients with eGFR below 60 ml/min/1.732 is the fear of further drop in their eGFR, despite the transient nature of this drop. All medications in this class are equally effective in providing glycemic control and other cardiovascular outcomes as demonstrated by many clinical trials [33,34]. Empagliflozin was the only SGLT2i available at the center of the study. This could be related to the cost-effectiveness of this medication compared to other medications [35]. Furthermore, this center is governmental. All dispensed medications are free, compared to private centers where the other types are available, and most patients dispense their medications through insurance companies.

SGLT2i use was also associated with better glycemic control than non-SGLT2i users in this cohort. The HbA1c level of more than 9% decreased from 5.9% to 7% of the cohort during the observation period among the SGLT2i users; however, both groups showed a significant reduction over nine months. SGLT2i have more than anti-glycemic effects; they have been associated with better blood pressure control and weight loss, which in turn can reduce insulin resistance in patients with type 2 diabetes [36,37].

Furthermore, a growing body of evidence demonstrates that the improved glycemic control with SGLT2i use could be related to the preservation of B-cell function and, hence, increased endogenous insulin secretion by reducing B-cell toxicity through SGLT2 inhibition [38,39]. The improvement in glycemic control among non-SGLT2i users cannot be explained fully from these data; however, using other antidiabetic medications plays an important role in attaining variable levels of HbA1c. This study also showed that patients with a high comorbidity index are more likely to have SGLT2i prescriptions, which is somewhat expected, given the better cardiovascular morbidity and mortality profile of this class of medications [12,16,40].

To the best of our knowledge, this is a rare study to describe the use of SGLT2i in Saudi Arabia. The strengths of this study include a long observation period of over five years and the use of multivariate analysis with adjustment for other confounders in addition to the assessment of glycemic control with the use of this class of medications. However, our study has some limitations. First, it was a single-center study; however, this center is considered the main referral site for all patients with diabetes from different hospitals in the region. Second, the relatively short follow-up period following the prescription may underestimate the glycemic control of SGLT2i among patients with diabetes. Finally, the lack of data on other antidiabetic medications could represent a confounding factor related to glycemic control; however, multivariate regression analysis has proved the strong relationship between SGLT2i use and better glycemic control.

## Conclusions

Our study showed that SGLT2i are being prescribed only in one-third of diabetic patients despite the availability, better glycemic control, and promising cardiovascular outcomes. Patients with uncontrolled diabetes mellitus, multiple visits to the clinics, and those without evidence of DKD are more likely to receive the prescription compared to their counterparts. Further studies are warranted to address the physician’s knowledge and perception about the prescription of this class of medication, in addition to the potential barriers to prescribe among different medical specialties.
